# Supplementary material for: Tumor-skin invasion is a reliable risk factor for poor prognosis in superficial soft tissue sarcomas
Source: PLoS One. 2022 Sep 2;17(9):e0274077. doi: 10.1371/journal.pone.0274077 (PMC9439222; doi:10.1371/journal.pone.0274077)
Supplement: S1 Table — (PDF) [file pone.0274077.s001.pdf]

Clinical information about cases

Case	Age	Sex	Final Pathological Diagnosis	Location	Size（cm）	Distant metastasis at initial diagnosis	Skin invasion	Outcome	Survival period(months)
1	69	M	Dedifferentiated liposarcoma	Chest	5	Negative	Negative	DOD	28
2	75	F	Epithelioid sarcoma	Neck	8	Negative	Negative	CDF	25
3	84	F	Dedifferentiated liposarcoma	Femur	5	Negative	Positive	DOD	23
4	81	F	Dedifferentiated liposarcoma	Lower leg	2	Negative	Negative	NED	111
5	82	M	Myxofibrosarcoma	Chest	5	Negative	Positive	DOAD	76
6	45	F	Synovial sarcoma	Foot	1	Negative	Negative	CDF	71
7	25	F	Solitary fibrous tumor	Abdomen	8	Negative	Negative	CDF	60
8	46	M	Ossifying fibromyxoid tumor	Upper arm	3	Negative	Negative	NED	54
9	87	M	Undifferentiated pleomorphic sarcoma	Thigh	11	Negative	Positive	AWD	21
10	79	M	Pleomorphic liposarcoma	Hip	3	Negative	Negative	DOD	3
11	78	F	Low grade fibromyxoid sarcoma	Lower leg	2	Negative	Negative	CDF	28
12	78	F	Atypical lipomatous tumor	Femur	10	Negative	Negative	DOAD	96
13	64	F	Pleomorphic liposarcoma	Femur	2	Negative	Negative	NED	144
14	37	M	Clear cell sarcoma	Femur	2	Negative	Negative	DOD	24
15	71	M	Low grade myxofibrosarcoma	Femur	3	Negative	Negative	DOD	89
16	71	F	Dedifferentiated liposarcoma	Forearm	3	Negative	Negative	NED	114
17	34	M	Dermatofibrosarcoma protuberans	Shoulder	3	Negative	Negative	NED	129
18	83	M	Leiomyosarcoma	Lower leg	4	Negative	Negative	CDF	34
19	80	F	Pleomorphic liposarcoma	Lower leg	5	Negative	Negative	CDF	115
20	79	F	Pleomorphic liposarcoma	Lower leg	2	Negative	Negative	NED	114
21	45	M	Myxoid liposarcoma	Femur	4	Negative	Negative	CDF	58
22	46	M	Epithelioid sarcoma	Abdomen	6	Positive	Positive	DOD	6
23	60	M	Malignant peripheral nerve sheath tumor	Lower leg	2	Negative	Negative	AWD	187
24	63	F	Myxoid liposarcoma	Femur	7	Negative	Negative	CDF	76
25	79	M	Myxoid liposarcoma	Femur	3	Negative	Negative	CDF	25
26	61	M	Solitary fibrous tumor	Femur	3	Negative	Negative	CDF	69
27	40	M	Epithelioid sarcoma	Hand	2	Negative	Negative	CDF	61
28	27	M	Epithelioid sarcoma	Forearm	10	Positive	Negative	DOD	144
29	41	M	Pleomorphic liposarcoma	Chest	10	Positive	Negative	CDF	152
30	57	M	Pleomorphic liposarcoma	Femur	6	Negative	Negative	DOD	21
31	67	M	Pleomorphic liposarcoma	Femur	16	Negative	Negative	CDF	72
32	71	F	Leiomyosarcoma	Forearm	8	Negative	Negative	NED	138
33	44	F	Myxoid liposarcoma	Femur	9	Negative	Negative	CDF	68
34	83	F	Leiomyosarcoma	Femur	2	Negative	Negative	DOD	45
35	83	M	Leiomyosarcoma	Shoulder	15	Positive	Negative	DOD	3
36	65	F	Myxofibrosarcoma	Forearm	5	Negative	Negative	NED	170
37	42	M	Synovial sarcoma	Foot	3	Negative	Negative	CDF	62
38	48	M	Dedifferentiated liposarcoma	Femur	10	Negative	Negative	CDF	145
39	57	M	Solitary fibrous tumor	Femur	8	Negative	Negative	NED	46
40	67	F	Myxofibrosarcoma	Abdomen	6	Positive	Negative	DOD	10
41	74	M	Myxofibrosarcoma (low grade)	Chest	3	Negative	Negative	CDF	50
42	71	F	Atypical lipomatous tumor	Shoulder	7	Negative	Negative	NED	71
43	80	M	Undifferentiated pleomorphic sarcoma	Chest	2	Negative	Negative	NED	70
44	55	M	Atypical lipomatous tumor	Shoulder	13	Negative	Negative	CDF	71
45	85	F	Leiomyosarcoma	Hip	3	Positive	Negative	NED	208
46	41	M	Undifferentiated pleomorphic sarcoma	Abdomen	7	Negative	Positive	DOD	48
47	21	F	Alveolar soft part sarcoma	Lower leg	7	Positive	Positive	DOD	9
48	61	M	Malignant peripheral nerve sheath tumor	Shoulder	7	Positive	Positive	DOD	34
49	67	M	Pleomorphic liposarcoma	Shoulder	12	Negative	Positive	DOAD	84
50	66	M	Pleomorphic liposarcoma	Upper arm	9	Positive	Positive	AWD	98
51	44	M	Pleomorphic liposarcoma	Chest	11	Negative	Positive	AWD	15
52	67	M	Undifferentiated pleomorphic sarcoma	Forearm	14	Positive	Positive	DOD	6
53	78	M	Pleomorphic liposarcoma	Chest	12	Negative	Positive	AWD	87
54	72	F	Atypical lipomatous tumor	Head	9	Negative	Negative	NED	59
55	85	F	Myxofibrosarcoma	Lower leg	3	Negative	Negative	NED	160
56	81	M	Atypical fibroxanthoma	Femur	4	Negative	Negative	CDF	56
57	84	F	Myxofibrosarcoma (low grade)	Lower leg	4	Negative	Negative	DOAD	10
58	76	M	Myxofibrosarcoma	Femur	10	Negative	Negative	CDF	14
59	79	F	Myxofibrosarcoma	Lower leg	5	Negative	Negative	DOD	12
60	50	F	Myxofibrosarcoma (low grade)	Upper arm	1	Negative	Negative	NED	43
61	84	F	Leiomyosarcoma	Upper arm	5	Negative	Negative	DOD	37
62	36	F	Undifferentiated pleomorphic sarcoma	Forearm	4	Negative	Negative	CDF	42
63	70	F	Atypical lipomatous tumor	Lower leg	3	Negative	Negative	CDF	42
64	73	F	Undifferentiated pleomorphic sarcoma	Femur	4	Negative	Negative	NED	45
65	74	F	Atypical lipomatous tumor	Shoulder	2	Negative	Negative	NED	65
66	71	M	Malignant peripheral nerve sheath tumor	Lower leg	7	Negative	Negative	AWD	55
67	72	F	Myxofibrosarcoma	Femur	5	Negative	Negative	NED	41
68	83	M	Undifferentiated pleomorphic sarcoma	Chest	3	Negative	Negative	NED	64
69	82	F	Dedifferentiated liposarcoma	Femur	2	Negative	Negative	CDF	35
70	58	M	MPNST	Forearm	3	Positive	Negative	AWD	88
71	71	M	Myxofibrosarcoma (low grade)	Lower leg	4	Negative	Negative	CDF	60
72	56	M	Dermatofibrosarcoma	Neck	7	Negative	Negative	CDF	35
73	49	M	Undifferentiated pleomorphic sarcoma (low grade)	Foot	3	Negative	Negative	NED	30
74	65	M	Undifferentiated pleomorphic sarcoma	Upper arm	9	Negative	Negative	AWD	31
75	76	M	Undifferentiated pleomorphic sarcoma	Chest	7	Negative	Negative	CDF	21
76	69	M	Atypical lipomatous tumor	Chest	29	Negative	Negative	CDF	18
77	75	M	Angiosarcoma	Forearm	3	Negative	Negative	AWD	23
78	53	M	Myxofibrosarcoma (low grade)	Forearm	3	Negative	Negative	CDF	19
79	78	F	Malignant giant cell tumour of soft tissue	Back	8	Positive	Negative	DOD	17
80	21	M	Low grade fibromyxoid sarcoma	Lower leg	4	Negative	Negative	CDF	19
81	27	M	Cellular angiofibroma	Femur	6	Negative	Negative	CDF	8
82	44	F	Solitary fibrous tumor	Femur	6	Negative	Negative	CDF	11
