# Supplementary material for: Tumor-skin invasion is a reliable risk factor for poor prognosis in superficial soft tissue sarcomas
Source: PLoS One. 2022 Sep 2;17(9):e0274077. doi: 10.1371/journal.pone.0274077 (PMC9439222; doi:10.1371/journal.pone.0274077)
Supplement: Attachment — Submitted filename: Response to Reviewers.docx [file pone.0274077.s002.docx]

**Journal Requirements:**

When submitting your revision, we need you to address these additional requirements.

1. Please ensure that your manuscript meets PLOS ONE's style requirements, including those for file naming. The PLOS ONE style templates can be found at
   <https://journals.plos.org/plosone/s/file?id=wjVg/PLOSOne_formatting_sample_main_body.pdf> and 
   <https://journals.plos.org/plosone/s/file?id=ba62/PLOSOne_formatting_sample_title_authors_affiliations.pdf>

**Response**: We thank the editor for these suggestions. We have gone through these templates and ensured that the style requirements of the PLOS ONE are met by making appropriate stylistic corrections in the revised manuscript.

2. In the ethics statement in the Methods and online submission information, please ensure that you have specified (1) whether consent was informed and (2) what type you obtained (for instance, written or verbal, and if verbal, how it was documented and witnessed). If your study included minors, state whether you obtained consent from parents or guardians. If the need for consent was waived by the ethics committee, please include this information.
   If you are reporting a retrospective study of medical records or archived samples, please ensure that you have discussed whether all data were fully anonymized before you accessed them and/or whether the IRB or ethics committee waived the requirement for informed consent. If patients provided informed written consent to have data from their medical records used in research, please include this information.

**Response**: We thank the editor for these suggestions. Our research was a retrospective study, which was approved by the Institutional Review Board of Osaka Metropolitan University Graduate School of Medicine and was performed in accordance with the ethical standards laid down in the Declaration of Helsinki (no. 4394). This information is a part of the methods section of the manuscript (Lines 80-84).

3. We note that you have indicated that data from this study are available upon request. PLOS only allows data to be available upon request if there are legal or ethical restrictions on sharing data publicly. For more information on unacceptable data access restrictions, please see <http://journals.plos.org/plosone/s/data-availability#loc-unacceptable-data-access-restrictions>.

In your revised cover letter, please address the following prompts:

a) If there are ethical or legal restrictions on sharing a de-identified data set, please explain them in detail (e.g., data contain potentially sensitive information, data are owned by a third-party organization, etc.) and who has imposed them (e.g., an ethics committee). Please also provide contact information for a data access committee, ethics committee, or other institutional body to which data requests may be sent.

b) If there are no restrictions, please upload the minimal anonymized data set necessary to replicate your study findings as either Supporting Information files or to a stable, public repository and provide us with the relevant URLs, DOIs, or accession numbers. For a list of acceptable repositories, please see <http://journals.plos.org/plosone/s/data-availability#loc-recommended-repositories>.

We will update your Data Availability statement on your behalf to reflect the information you provide.

**Response**: We thank the editor for these insights. We have added and uploaded the minimal anonymized data set as supporting information (Page 26, lines 455-456).

4. Please upload a copy of Figures 2 and 3 to which you refer in your text on pages 7 and 15. If the figure is no longer to be included as part of the submission please remove all reference to it within the text.

**Response**: We thank the editor for pointing this out. We have uploaded the copies of Figures 2 and 3 in the revised submission.

5. Please review your reference list to ensure that it is complete and correct. If you have cited papers that have been retracted, please include the rationale for doing so in the manuscript text, or remove these references and replace them with relevant current references. Any changes to the reference list should be mentioned in the rebuttal letter that accompanies your revised manuscript. If you need to cite a retracted article, indicate the article’s retracted status in the References list and also include a citation and full reference for the retraction notice.

**Response**: We thank the editor for their advice. We have rechecked our reference list and corrected that in the revised manuscript.

**Review Comments to the Author**

**Reviewer #1**: I do not have many suggestions regarding the paper itself, it is quite well written, with good literature review.
The quality of figures should be improved.

**Response**: We thank the reviewer for evaluating our manuscript and for these valuable suggestions. We have improved the quality of figures and uploaded them in the revised submission.

I would suggest to cite this recent review: Oettel, D.J., Bernard, S.A. Review of primary superficial soft tissue mesenchymal tumors of malignant or intermediate biological potential. Skeletal Radiol (2022). <https://doi.org/10.1007/s00256-022-04127-0>

**Response**: We thank the reviewer for their advice. We believe that citing this review has indeed improved our manuscript. We have cited this article on Page 4, lines 46-48.

Page 4, lines 46-48: However, when the tumors occur superficially, that might distort the clinical decision-making of the oncologists and thus, they might not suspect sarcomas, resulting in a misdiagnosis [5].

**Reviewer #2**: In the manuscript entitled “Tumor-skin Invasion is a Reliable Risk Factor for Poor Prognosis in Superficial Soft Tissue Sarcomas” the authors describe that the tumor-skin invasion is closely associated with poor prognoses.

The manuscript is well written and the data are presented with great accuracy. However, I have some minor suggestions to share with the authors:

• I suggest the authors provide the statistical analysis used in the legend of tables.

**Response**: We thank the reviewer for a careful review of our manuscript and for their helpful suggestions. We have added the statistical analysis used in the legend of tables. Please refer to Table 3a (Cox regression analysis) and Table 3b (Fisher’s exact probability test).

• The "Introduction" section is poor in information. the authors have to expand the text with the immunological aspect of sarcoma. For this aspect, I suggest citing the article "Sarcoma Common MHC-I Haplotype Restricts Tumor-Specific CD8+ T Cell Response. 2022, doi.org/10.3390/cancers14143414".

**Response**: We thank the reviewer for highlighting this gap and for suggesting us the remedy. We believe that including this citation has certainly strengthened the manuscript. We have cited this article on Page 4, lines 58-60.

Page 4, lines 58-60: even though cutting-edge research indicates that an assessment of the major histocompatibility complex is crucial for the clinical outcome of sarcoma immunotherapy [10].

• In addition, the authors could discuss the mechanisms of invasion.

**Response**: We thank the reviewer for this kind advice. We have added the details and believe that this has helped us to improve the manuscript (Page 21, lines 335-338).

Page 21, lines 335-338: Fourth, we did not examine the immunological and molecular mechanisms of tumor-skin invasion. Therefore, it will be indispensable for further research to elucidate the relationship between invasion and poor prognosis in the future.

• Finally, the future perspectives of the authors must be more elaborated.

**Response**: We thank the reviewer for these insights. We have included future perspectives based on the reviewer’s suggestions (Page 21, lines 351-354).

Page 21, lines 351-354: Not only further prospective studies enrolling a larger number of patients and involving multiple centers but also strategic basic research, such as immunological and molecular evaluation is warranted to substantiate the relationship between tumor-skin invasion, distant metastasis, and poor prognosis.

• English revision is required.

**Response**: We thank the reviewer for this helpful advice. We have substantially improved the quality of English used in our manuscript.
